# Indirect Comparison Showed Survival Benefit from Adjuvant Chemoradiotherapy in Completely Resected Gastric Cancer with D2 Lymphadenectomy

**DOI:** 10.1155/2013/634929

**Published:** 2013-10-01

**Authors:** Qiong Yang, Ying Wei, Yan-Xian Chen, Si-Wei Zhou, Zhi-Min Jiang, De-Rong Xie

**Affiliations:** ^1^Department of Oncology, Sun Yat-sen Memorial Hospital, Sun Yat-sen University, Guangzhou 510120, China; ^2^Department of Oncology, Huizhou Center Hospital, Huizhou 516001, China

## Abstract

*Background*. Little data on directly comparing chemoradiotherapy with observation has yet been published in the setting of adjuvant therapy for resected gastric cancer who underwent D2 lymphadenectomy. The present indirect comparison aims to provide more evidence on comparing the two approaches. *Methods*. We conducted a systematic review of randomized controlled trials, extracted time-to-event data using Tierney methods (when not reported), and performed indirect comparison to obtain the relative hazards of adjuvant chemoradiotherapy to observation on overall and disease-free survival. *Results*. seven randomized controlled trials were identified. Three trials compared adjuvant chemoradiotherapy with adjuvant chemotherapy, and 4 trials compared adjuvant chemotherapy with observation. Using indirect comparison, the relative hazards of adjuvant chemoradiotherapy to observation were 0.43 (95% CI: 0.33–0.55) in disease-free survival and 0.52 (95% CI: 0.38–0.71) in overall survival for completely resected gastric cancer with D2 lymphadenectomy. *Conclusions*. Postoperative chemoradiotherapy can prolong survival and decrease recurrence in patients with resected gastric cancer who underwent D2 gastrectomy. Molecular biomarker might be a promising direction in the prediction of clinical outcome to postoperative chemoradiotherapy, which warranted further study.

## 1. Introduction

Gastric cancer is the third leading cause of cancer-related death among men and the fifth among women in the worldwide [1]. The primary curative treatment of gastric carcinoma is surgical resection [2]. Complete resection with adequate margins is widely considered as a standard goal, whereas the extent of lymph node dissection remains controversial. Irrespective of the surgical procedure used for the treatment of gastric cancer, the effectiveness of surgical resection is poor; about 60% eventually have local relapse or distant metastases after curative resection [3]. The high rate of relapse or distant metastases after resection make it important to consider adjuvant treatment for patients with resected gastric cancer. 

The INT-0116 trial [4, 5], the largest phase III trial comparing chemoradiotherapy versus observation, shows that adjuvant chemoradiotherapy prolonged overall survival (OS) and relapse-free survival (RFS). In this trial, 10% of the patients underwent D2 dissection, suggesting that chemoradiotherapy might be only compensating for inadequate surgery. Therefore, the role of chemoradiation therapy after D2 dissection has been questioned. Two retrospective studies demonstrated that adjuvant chemoradiotherapy was well tolerated with acceptable toxicities and reasonable tumor control for patients with D2 gastrectomy [6, 7]. Another retrospective study does not demonstrate that adjuvant chemoradiotherapy reduce relapse and impact on survival [8]. There is no RCT comparing adjuvant chemoradiotherapy with observation D2-dissected gastric cancer. In view of the paucity of data, we attempted to answer this question using the method of adjusted indirect comparison.

## 2. Methods

### 2.1. Literature Search

A systematic review of eligible RCTs was performed by searching the electronic databases, which consist of Cochrane Central Register of Controlled Trials, Medline, EMBASE, ISI Web of Knowledge, ASCO abstracts, and ESMO abstracts. The deadline of this search was June 30, 2013. The keywords were used for search in electronic databases as follows: “gastric cancer,” “Stomach Neoplasms,” “chemoradiotherapy,” “chemoradiation,” “Chemotherapy,” “D2”, and “Combined Modality Therapy.” The search was limited to RCTs in English language. The reference lists of articles were identified, and relevant meta-analysis were searched manually to find other relevant articles. 

### 2.2. Trial Selection and Quality Assessment

All RCTs that compared chemotherapy with observation or compared chemoradiotherapy with chemotherapy in adjuvant therapy for resected gastric cancer were included in the present study. If the same population appeared in other publications, the article that provided the most complete follow-up data on survival was selected. Methodological quality of the trials was assessed using a validated scale (range, 0 to 5) applied to items that influence intervention efficacy. The scale consists of items pertaining to randomization, masking, dropouts, and withdrawals, which is reported by Jadad et al. [9]. Trial was regarded as high quality trial and had high external and internal validities if it was scored by more than 3 points.

### 2.3. Data Extraction

Two primary reviewers assessed all abstracts that were identified from the above-mentioned sources. Both reviewers independently selected potentially eligible abstracts according to inclusion criteria. If one of the reviewers concluded an abstract that might be eligible, the complete article was retrieved and reviewed in detail by both reviewers. Disagreements were resolved by consensus or by the third reviewer. Hazard ratio (HR) and 95% confidence interval (95% CI) for OS and DFS were requested. Where published, HR and 95% CI were extracted directly from the original article. Where HR and 95% CI were not reported, they were calculated from published summary statistics or survival curve using Tierney et al. method [10]. The following variables were extracted from each trial if available: first author's name, publication year, country of origin, treatment regimen, total numbers of patients, percentage of different stages, percentage of T3 and T4 stage, percentage of lymph node positive, HR and 95% CI for OS and DFS, and median follow-up time.

### 2.4. Brief Introduction of Adjusted Indirect Comparison

Suppose that interventions A and C were directly compared in a RCT, and another trial compared intervention B with intervention C. To compare intervention A with intervention B, a method of adjusted indirect comparison can be used to realize it [11]. Briefly, the log hazard ratio (log HR) of the adjusted indirect comparison for intervention A versus B was estimated by log⁡ HR_AB_ = log⁡ HR_AC⁡_ − log⁡ HR_BC_, and its standard error for the log HR was SE$(\log\;{\text{HR}}_{\text{AB}})=\sqrt{\text{SE}{\left(\log\;\text{H}{\text{R}}_{\mathrm{AC}}\right)}^{2}+\text{SE}{\left(\log\;\text{H}{\text{R}}_{\text{BC}}\right)}^{2}}$. 

Where log⁡ HR_AC⁡_ was the log HR for the direct comparison of intervention A versus C and log⁡ HR_BC_ were the log HR for the direct comparison of intervention B versus C. SE(log⁡ HR_AC⁡_) was the standard error of the log⁡ HR for the direct comparison of intervention A versus C and SE(log⁡ HR_BC_) was the standard error of the log HR for the direct comparison of intervention B versus C. The strong underlying assumption in this adjusted indirect comparison method is that the relative efficacy of an intervention is consistent in patients included in different trials. That is, log⁡ HR_AC⁡_ observed in trials comparing A versus C is assumed to be log⁡ HR_BC_ that would have been observed in those trials comparing B versus C and vice versa. 

### 2.5. Statistical Analysis

To combine the results of individual trial's HR for comparing chemoradiotherapy with chemotherapy or comparing observation with chemotherapy, direct meta-analysis was used. Heterogeneity assumption was checked by a chi-square-based *Q*-test and also expressed as *I* ^2^. A *P* value of more than 0.10 for the *Q*-test and *I* ^2^ of less than 50% indicated a lack of heterogeneity across the trials. If *P*-value of heterogeneity test was more than 0.1 and *I* ^2^ was less than 50%, fixed effects model was performed and random effects model was used vice versa. 

Adjusted indirect comparison was used to evaluate the relative efficacy of chemoradiotherapy to observation. The primary end point was OS, and the secondary end point was DFS. Treatment effect size was calculated by HR and 95% CI. Due to the adjusted indirect comparison using the fixed effect model which tended to underestimate standard errors of pooled estimates, random effect model was used for the quantitative pooling in the adjusted indirect comparison. A HR value of less than 1 stands for favoring chemoradiotherapy arm and a HR value of more than 1 stands for favoring chemotherapy arm. All CIs had a two-sided probability coverage of 95%. A statistical test with a *P* value less than 0.05 was considered significant, and all *P* values were two-sided. 

All analyses were performed strictly with RevMan software (version 5.2, Cochrane).

## 3. Results

### 3.1. Trial Flow, Characteristics, and Quality Appraisal

**Figure 1:**
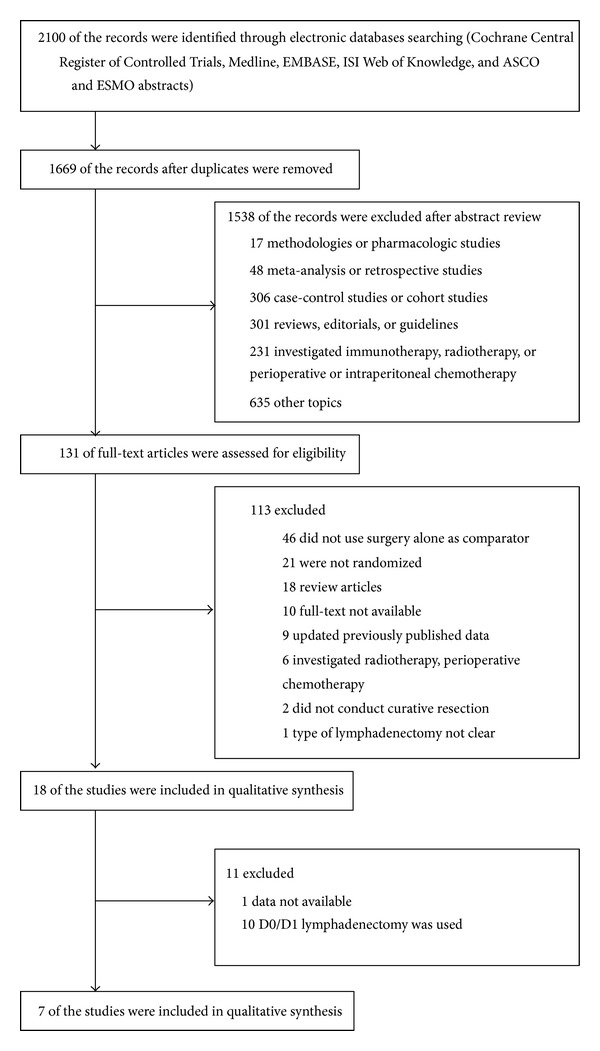
Flow chart of randomized controlled trials selection.

Figure 1 was the flow chart of RCTs selection for this study. Seven trials were identified at last [12–18]. Three trials compared chemoradiotherapy with chemotherapy. Four trials directly compared observation with chemotherapy. Six out of 7 trials were conducted in Asian countries, but only one trial was from European country. Almost all patients (>95%) underwent curative gastrectomy with D2 lymphadenectomy.  Table 1 showed important baseline characteristics and Jadad scores of selected trials. 

### 3.2. Adjusted Indirect Comparison

First, we use a method of meta-analysis to combine the pooled result for chemoradiotherapy versus chemotherapy and observation versus chemotherapy. The pooled HR and 95% CI were 0.72 (0.59–0.89) in DFS and 0.79 (0.61–1.03) in OS for chemoradiotherapy versus chemotherapy. The pooled HR and 95% CI were 1.68 (1.46–1.93) in DFS and 1.52 (1.30–1.79) in OS for observation versus chemotherapy. 

Second, adjusted indirect comparison was computed for estimating the relative efficacy of adjuvant chemoradiotherapy to observation. Compared with observation, chemoradiotherapy significantly improved DFS and OS for patients with D2-resected gastric cancer. The pooled HR and 95% CI were 0.43 (0.33–0.55) in DFS and 0.52 (0.38–0.71) in OS, respectively.  Table 2 summarized those estimates of indirect comparison for D2-resected gastric cancer.

### 3.3. Subgroup Analyses

To explore the potential influence on survival benefit by geographic difference, we reevaluated the pooled HR for observation to chemotherapy by omitting the trial from Spain and did further indirect comparison of chemoradiotherapy to observation. The pooled HR for observation to chemotherapy was 1.66 (1.44–1.92) in DFS and 1.50 (1.26–1.78) in OS, respectively. Accordingly, the pooled HR for indirect comparison of chemoradiotherapy to observation was 0.43 (0.34–0.56) in DFS and 0.53 (0.38–0.72) in OS, respectively (Table 2). 

## 4. Discussion

In the last decade, postoperative chemoradiotherapy has become the preferred strategy for resected gastric cancer in the United States because the INT-0116 trial suggested that postoperative chemoradiotherapy had a survival advantage over observation. However, adoption of this regimen has been somewhat tempered in Asian countries. The main reason was the inadequate node dissection (only 10% had a D2 dissection) in INT-0116. Recently, gastrectomy with D2 lymphadenectomy becomes the standard treatment for curable gastric cancer in Eastern Asia. Thus, the efficacy of adjuvant chemoradiotherapy should be established in patients with D2-resected gastric cancer.

To evaluate the relative efficacy of treatment approaches, the most reliable evidence comes from head-to-head RCTs. However, there is usually no direct randomized evidence or no sufficient direct randomized evidence. In this situation, adjusted indirect comparison of different interventions can be used to give an alternative estimation. It is reported that results of adjusted indirect comparison usually, but not always, agree with those of head to head randomized trials [19]. Due to insufficient direct evidence, we used an adjusted indirect comparison method to estimate the efficacy of adjuvant chemoradiotherapy to observation in completely resected gastric cancer. Overall, our data demonstrated strong benefit from adjuvant chemoradiotherapy in patients with D2-resected gastric cancer.

A Singapore retrospective study reports the clinical outcomes of 67 patients who were mostly treated with D2 node dissection and received adjuvant chemoradiotherapy as per INT-0116. The 3-year overall survival, disease-free survival, and local control are 60.6%, 54.1%, and 84.3%, respectively. Of the 30 patients who relapsed, 5 (17%) have isolated locoregional recurrences only. This retrospective study shows reasonable tumor control benefit from adjuvant chemoradiotherapy [6]. Comparable results were also showed in a Korean retrospective observational study with over 500 cases after D2 gastrectomy [7]. On the other hand, 3-year overall survival, disease-free survival, and local control in chemotherapy arm are 80.1%, 72.2%, and 75% in ACTS-GC trial, respectively [17]. Similarly, 3-year disease-free survival and local control in chemotherapy arm are 74% and 82% in CLASSIC trial, respectively [18]. Although simple horizontal comparison is unscientific, it seemed that chemoradiotherapy arm of two retrospective studies did not show an advantage over chemotherapy arm of ACTS-GC and CLASSIC trials. To date, 3 head-to-head trials compare chemoradiotherapy versus chemotherapy for those patients without positive results reported [12–14]. Furthermore, pooled analysis of these 3 trials also does not demonstrate that chemoradiotherapy has any survival advantage over chemotherapy [20]. 

The reason that chemoradiotherapy did not have any survival advantage over chemotherapy in D2-resected gastric cancer was not well understood. It has been reported that the sites of treatment failure after surgical treatment were mainly locoregional in the tumor bed in Western countries [21]. In contrast, in Asian countries, the sites of treatment failure were mainly distant metastasis [20]. The discrepancy is mainly due to a high percentage of diffuse-type histology gastric cancer in Asian population, which accounted for 50% at least [22]. Diffuse gastric cancer is characterized by decreased intracellular adhesion as a result of E-cadherin mutation and/or hypermethylation and is prone to early metastasis. Therefore, chemoradiotherapy does not appear to confer a benefit to diffuse gastric cancer [23]. 

There are studies to explore the role of molecular biomarkers in predicting clinical outcome to chemoradiotherapy. A study evaluates the potential association of xeroderma pigmentosum group D (XPD) codon 751 variant with outcome after chemoradiotherapy in 44 patients with resected gastric cancer. It indicates that 75% of relapse patients show Lys/Lys genotype more frequently (*P* = 0.042). The Lys polymorphism is an independent predictor of high-risk relapse-free survival from statistical analysis (HR: 3.07, 95% CI: 1.07–8.78, *P* = 0.036) [24]. Recently, INT-0116 group reports result of a retrospective analysis on the prognostic value of HER2 in adjuvant therapy choice for gastric cancer. Patients are from INT-0116 phase III gastric cancer clinical trial. Among patients with HER2-nonamplified cancers, treated patients have a median OS of 44 months compared with 24 months in the surgery-only arm (*P* = 0.003). Among patients with HER2-amplified cancers, there is no significant difference in survival based on treatment arm. HER2 status is not a prognostic marker among patients who received no postoperative chemoradiotherapy [25]. In short, molecular biomarkers might be a promising direction to screen the patients who benefit from postoperative chemoradiotherapy, which warranted further study.

There were several important limitations to our study. First, patient characteristics might be different among selected trials. Cancer stage in the majority of trials was at more advanced stage, including stage II/III, T3/4, and N+ patients, in contrast to the greater proportion of stage I and less proportion of T3/4 patients included in the Nashimoto et al. trial. However, the result of adjusted indirect comparison was not materially altered after omitting this trial (data not shown). Second, because only the published literature and English literature were reviewed for the study, there is the potential for results to be influenced by publication bias and selection bias. Third, the treatment protocols among included RCTs were different with each other. However, due to limited number of the final included RCTs, we did not perform subgroup analysis based on treatment protocols. That is, head to head comparison is needed urgently in future. At last, 6 out of 7 eligible trials were from Asia, making the result less generalized to other region. 

## 5. Conclusions

Based on indirect comparison, chemoradiotherapy demonstrated strong survival advantage over observation in patients with D2-resected gastric cancer. We confirmed the role of adjuvant chemoradiotherapy in D2-resected gastric cancer patients from a different perspective. At present, there are studies which reported that molecular biomarkers might predict clinical outcome to chemoradiotherapy, which was helpful to develop individualized therapy and warranted further study. 

## Figures and Tables

**Table 1:** Characteristics of selected RCTs.

References	Countries	Regimens	*N*	I/II/III/IV (%)	T3/4 (%)	N+ (%)	DFS	OS	Median (range)	Jadad score
							HR (95% CI)	HR (95% CI)	Follow-up (month)	
Chemoradiotherapy versus chemotherapy										
Lee et al. 2012 [12]	Korea	XP/XRT/XP	230	21.3/36.5/30.8/11.3	NR	88.3	0.73 (0.52–1.04)	—	53.2 (36.9–77.3)	3
		XP	228	21.9/37.7/28.6/11.8	NR	84.6				
Zhu et al. 2012 [13]	China	RT/FL	168	10.8/19.4/55.4/14.5	100	84.9	0.74 (0.56–0.97)	0.80 (0.6–1.06)	42.5	3
		FL	165	9.1/18.2/58.2/14.5	100	86.7				
Kim et al. 2012 [14]	Korea	RT/FL	46	0/0/73.9/26.1	69.5	100	0.62 (0.33–1.14)	0.76 (0.39–1.48)	86.7 (60.3–116.5)	3
		FL	44	0/0/75/25	56.8	95.4				

Observation versus chemotherapy										
Cirera et al. 1999 [15]	Spain	MMC/tegafur	76	0/0/100/0	92	80	1.82 (1.18, 2.79)	1.67 (1.08, 2.57)	37 (3–122)	4
		Observation	72	0/0/100/0	89	97				
Nakajima et al. 2007 [16]	Japan	Uracil-tegafur	93	0/100/0/0	0	100	2.27 (1.28, 1.04)	2.08 (1.13, 3.85)	74.4	5
		Observation	95	0/100/0/0	0	100				
Sasako et al. 2011 [17]	Japan	S-1	529	0.2/49.9/42.3/7.6	45.1	90.4	1.54 (1.27, 1.86)	1.49 (1.20, 1.85)	60	3
		Observation	530	0/53.2/40.2/6.6	46.1	87.9				
Bang et al. 2012 [18]	Korea	XELOX	520	1/49/51/0	45	91	1.79 (1.40, 2.28)	1.39 (1.00, 1.93)	34.2 (25.4–41.7)	3
		Observation	515	0/51/49/0	45	89				

RCTs: randomized controlled trials; *N*: number of patients; I/II/III/IV: cancer stage; IV refers to T4N1-3M0 and T1-3N3M0; T3/4: percentage of T3 and T4 stage; N+: percentage of node positive; DFS: disease-free survival; OS: overall survival; HR: hazard ratio; 95% CI: 95% confidence interval.

NR: not report.

XP: capecitabine + cisplatin; XRT: radiotherapy with capecitabine; RT: radiotherapy; FL: fluorouracil plus leucovorin; MMC: mitomycin; XELOX: oxaliplatin + capecitabine.

**Table 2:** Indirect comparison on the efficacy of chemoradiotherapy versus observation for resected gastric cancer after D2 lymphadenectomy.

CRT versus Obs	Number of trials in comparison	Hazard ratio (95% CI)	*P* value
DFS	7	0.43 (0.33, 0.55)	0.00
	6*	0.43 (0.34, 0.56)	0.00
OS	6	0.52 (0.38, 0.71)	0.00
	5*	0.53 (0.38, 0.72)	0.00

CRT: chemoradiotherapy, Obs: observation, 95% CI: 95% confidence interval, DFS: disease-free survival, and OS: overall survival. *Subgroup analysis after omitting the trial from Spain.
